# Pan-cancer analysis of Krüppel-like factor 3 and its carcinogenesis in pancreatic cancer

**DOI:** 10.3389/fimmu.2023.1167018

**Published:** 2023-08-03

**Authors:** Jinfeng Zhu, Hong Teng, Xiaojian Zhu, Jingxuan Yuan, Qiong Zhang, Yeqing Zou

**Affiliations:** ^1^ Jiangxi Province Key Laboratory of Molecular Medicine, The Second Affiliated Hospital of Nanchang University, Nanchang, Jiangxi, China; ^2^ Department of Medical Genetics, The Second Affiliated Hospital of Nanchang University, Nanchang, China; ^3^ School of Public Health, Nanchang University, Nanchang, Jiangxi, China; ^4^ Tomas Lindahl Nobel Laureate Laboratory, The Seventh Affiliated Hospital of Sun Yat-sen University, Shenzhen, China

**Keywords:** *KLF3*, pan-cancer, prognosis, immunotherapy, tumor microenvironment, pancreatic cancer

## Abstract

**Background:**

Krüppel-like factor 3 (*KLF3*) is a key transcriptional repressor, which is involved in various biological functions such as lipogenesis, erythropoiesis, and B cell development, and has become one of the current research hotspots. However, the role of *KLF3* in the pan-cancer and tumor microenvironment remains unclear.

**Methods:**

TCGA and GTEx databases were used to evaluate the expression difference of *KLF3* in pan-cancer and normal tissues. The cBioPortal database and the GSCALite platform analyzed the genetic variation and methylation modification of *KLF3*. The prognostic role of *KLF3* in pan-cancer was identified using Cox regression and Kaplan-Meier analysis. Correlation analysis was used to explore the relationship between *KLF3* expression and tumor mutation burden, microsatellite instability, and immune-related genes. The relationship between *KLF3* expression and tumor immune microenvironment was calculated by ESTIMATE, EPIC, and MCPCOUNTER algorithms. TISCH and CancerSEA databases analyzed the expression distribution and function of *KLF3* in the tumor microenvironment. TIDE, GDSC, and CTRP databases evaluated *KLF3*-predicted immunotherapy response and sensitivity to small molecule drugs. Finally, we analyzed the role of *KLF3* in pancreatic cancer by *in vivo* and *in vitro* experiments.

**Results:**

*KLF3* was abnormally expressed in a variety of tumors, which could effectively predict the prognosis of patients, and it was most obvious in pancreatic cancer. Further experiments verified that silencing *KLF3* expression inhibited pancreatic cancer progression. Functional analysis and gene set enrichment analysis found that *KLF3* was involved in various immune-related pathways and tumor progression-related pathways. In addition, based on single-cell sequencing analysis, it was found that *KLF3* was mainly expressed in CD4Tconv, CD8T, monocytes/macrophages, endothelial cells, and malignant cells in most of the tumor microenvironment. Finally, we assessed the value of *KLF3* in predicting response to immunotherapy and predicted a series of sensitive drugs targeting *KLF3*.

**Conclusion:**

The role of *KLF3* in the tumor microenvironment of various types of tumors cannot be underestimated, and it has significant potential as a biomarker for predicting the response to immunotherapy. In particular, it plays an important role in the progression of pancreatic cancer.

## 1 Introduction

In the world, cancer is the second most common cause of death, accounting for one in six deaths (1). In 2022, there were 1,918,030 new cancer cases, and 609,360 cancer-related deaths in the USA, according to the report on cancer statistics (2). Despite years of sustained effort, the long-term results of treatments using traditional strategies remain dismal. A major obstacle limiting the effectiveness of conventional cancer therapies was their tumor specificity (3). In recent years, tumor immunotherapy has received increasing attention, including immune checkpoint blockade therapy, immune cell therapy, and tumor vaccine therapy (4, 5). The specificity of immunotherapy depends largely on the specific tumor antigen (6). However, immunotherapy-related biomarker matching trials were still limited in most cancers (7). Therefore, further exploration of effective immunotherapy-related tumor prognostic biomarkers is urgently needed.

Krüppel-like factor (KLF) 3 is a member of the KLF transcription factor family, which is involved in various physiological processes such as adipogenesis, erythrocyte maturation, B cell differentiation, and cardiovascular development (8). *KLF3* also has a special zinc finger structure, which can bind to related CACCC elements to regulate the expression of target genes, thereby regulating cell proliferation, migration, and apoptosis, and it is also critical to early embryonic development (9). In recent years, studies have found that *KLF3*, as a transcriptional repressor, is abnormally expressed in a variety of tumors, including colon cancer (10), breast cancer (11), lung cancer (12), pancreatic cancer (13), etc. *KLF3* plays an important role in different tumor types. For example, studies have shown that *KLF3* becomes a key regulator of metastasis by controlling the expression of *STAT3* in lung cancer, and silencing *KLF3* promote lung cancer EMT and enhances lung cancer metastasis (14); Another study showed that miR-365a-3p targets *KLF3* to inhibit colorectal cancer cell migration, invasion and chemotherapy resistance (15). Tian et al. reported that miR-660-5p-loaded M2 macrophage-derived exosomes promoted the development of hepatocellular carcinoma by regulating *KLF3* (16). In addition, Zhang et al. also found that aberrant expression of *KLF3* was associated with acquired resistance to fluorouracil in colon cancer cells (17). However, the expression levels and clinical significance of *KLF3* in most cancer types remain to be elucidated.

In this study, a comprehensive bioinformatics analysis of *KLF3* was conducted through multiple databases to clarify the expression, abnormal variation, and clinical significance of *KLF3* in pan-cancer. The role of *KLF3* in the tumor immune microenvironment was further analyzed, and the relationship between *KLF3* and immunotherapy response and related sensitive drugs was evaluated. We also focused on analyzing the relationship between *KLF3* abnormal expression and pancreatic cancer progression using *in vitro* and *in vivo* experiments, and identified *KLF3* as an independent prognostic risk factor for pancreatic cancer.

## 2 Materials and methods

### 2.1 Pan-cancer data collection

We organize the pan-cancer data through the TCGA database and standardize the data to log2 (TPM + 1), which is used for the differential analysis of *KLF3* gene expression between paired normal tissues and cancer tissues, draw Kaplan-Meier curves for survival analysis and independent prognostic analysis, etc. In addition, the normalized pan-cancer dataset from TCGA TARGET GTEx (PANCAN, N=19131, G=60499) was downloaded from the UCSC (https://xenabrowser.net/) database. *KLF3* gene differential expression analysis of unpaired normal and cancer tissues, clinical feature correlation analysis, Cox prognosis analysis, and immune feature correlation analysis were performed by SangerBox (18), and the parameter selection sequencing data were normalized to log2 (x + 1). In **Table 1**, we report the abbreviation for each tumor type.

**Table 1:** Tumor types and abbreviations.

Abbreviation	Full name
ACC	Adrenocortical carcinoma
ALL	Acute Lymphoblastic Leukemia
AML	Acute myeloid leukemia
AST	Astrocytoma
BLCA	Bladder Urothelial Carcinoma
BRCA	Breast invasive carcinoma
CESC	Cervical squamous cell carcinoma and endocervical adenocarcinoma
CHOL	Cholangiocarcinoma
CML	Chronic myelogenous leukemia
COAD	Colon adenocarcinoma
COADREAD	Colon adenocarcinoma/Rectum adenocarcinoma Esophageal carcinoma
ESCA	Esophageal carcinoma
GBM	Glioblastoma multiforme
GBMLGG	Glioma
HGG	High-grade glioma
HNSC/HNSCC	Head and Neck squamous cell carcinoma
KICH	Kidney Chromophobe
KIPAN	Pan-kidney cohort (KICH+KIRC+KIRP)
KIRC	Kidney renal clear cell carcinoma
KIRP	Kidney renal papillary cell carcinoma
LAML	Acute Myeloid Leukemia
LGG	Brain Lower Grade Glioma
LIHC	Liver hepatocellular carcinoma
LUAD	Lung adenocarcinoma
LUSC	Lung squamous cell carcinoma
MEL	Melanoma
NSCLC	Non-small cell lung cancer
ODG	Oligodendroglioma
OV	Ovarian serous cystadenocarcinoma
PAAD	Pancreatic adenocarcinoma
PCPG	Pheochromocytoma and Paraganglioma
PRAD	Prostate adenocarcinoma
RB	Retinoblastoma
RCC	Renal cell carcinoma
READ	Rectum adenocarcinoma
SARC	Sarcoma
SKCM	Skin Cutaneous Melanoma
STAD	Stomach adenocarcinoma
STES	Stomach and Esophageal carcinoma
TGCT	Testicular Germ Cell Tumors
THCA	Thyroid carcinoma
THYM	Thymoma
UCEC	Uterine Corpus Endometrial Carcinoma
UCS	Uterine Carcinosarcoma
UM	Uveal Melanoma
UVM	Uveal Melanoma
WT	High-Risk Wilms Tumo

### 2.2 Prognostic analysis of *KLF3* in pan-cancer

To clarify the effect of *KLF3* on the prognosis of tumor patients, Cox proportional hazards regression mode (19) was established to analyze the correlation between *KLF3* expression and the overall survival (OS), disease-specific survival (DSS), disease-free interval (DFI) and progression-free interval (PFI) of each cancer type. The “surv_cutpoint” function in the “survminer” package was utilized to perform an optimal cut-off selection for distinguishing between high and low expression groups. Followed by a Log-rank test for analyzing the survival differences, and the results were visualized using both “survminer” and “ggplot2” packages.

### 2.3 *KLF3* protein expression and localization

We obtained the protein expression difference of the *KLF3* between pancreatic cancer and normal pancreatic tissue by querying the UALCAN (20) database. Additionally, the subcellular localization of the *KLF3* gene was determined using the human gene database Genecards (https://www.genecards.org/).

### 2.4 *KLF3* genetic alteration, methylation, and RNA modification analysis

Access the cBioPortal database online (http://www.cbioportal.org/), and select the “TCGA PanCancer Atlas Studies”, “*KLF3*” and “Cancer Types Summary” modules to obtain information on the genomic alteration types and frequencies of *KLF3* in pan-cancer. Online access to Gene Set Cancer Analysis (GSCA, http://bioinfo.life.hust.edu.cn/GSCA/#/), based on Spearman correlation analysis *KLF3* expression of gene copy number variation (CNV) and methylation. The DNA methylation levels of the *KLF3* gene promoter were from the UALCAN database (http://ualcan.path.uab.edu). Finally, the correlation of *KLF3* with 3 types of RNA modifications (m1A (10), m5C (13), and m6A (21)) was analyzed using Spearman’s correlation analysis, and the results were visualized using SangerBox tools.

### 2.5 The function and enrichment analysis

To identify differentially expressed genes between low and high *KLF3* subgroups in each cancer type, patients were ranked based on their *KLF3* expression levels. The top 30% of patients were classified as the high *KLF3* subgroup, while the bottom 30% were classified as the low *KLF3* subgroup. The “limma” R package was employed for analyzing *KLF3*-related differentially expressed genes in each cancer type, considering an adjusted p-value threshold of <0.05. Gene set enrichment analysis was performed using the R packages “clusterProfiler” (21) and “GSVA” (22). The annotated gene set (h.all.v7.2.symbols.gmt) was selected as the reference gene set for enrichment analysis. The pan-cancer Normalized Enrichment Score (NES) and False Discovery Rate (FDR) were calculated for each biological process. The results were visualized using the “ggplot2” R package in the form of a bubble plot.

Moreover, we accessed the Cancer Single-cell State Atlas (CancerSEA, biocc.hrbmu.edu.cn/CancerSEA/home.jsp) database and conducted an analysis of the single-cell RNA sequencing data by specifically examining the gene “*KLF3*”. This analysis allowed us to uncover the intricate relationship between *KLF3* gene expression and the diverse repertoire of 14 distinct states observed within cancer.

### 2.6 Immune cell infiltration analysis and large-scale single-cell data sequencing validation

To conduct a reliable immune correlation assessment, we used the MCP-counter (23) and EPIC (24) algorithms to calculate the Spearman’s correlation coefficient between the *KLF3* gene and immune cell infiltration in each tumor and presented the results in the form of a heat map.

Online access to Tumor Immune Single-cell Hub 2 (TISCH2, http://tisch.comp-genomics.org/home/), select datasets: ALL-GSE132509, BLCA-GSE130001, BRCA-GSE138536, CHOL-GSE125449, CRC-GSE139555, Glioma-GSE103224, HNSC-GSE103322, KIRC-GSE111360, LIHC-GSE140228, NSCLC-EMTAB6149, OV-GSE118828, PAAD-CRA001160, SKCM-GSE123139, STAD-GSE134520, UCEC-GSE139555 and UVM-GSE139829. The gene “*KLF3*” was further input for single-cell sequence data analysis to clarify the expression level of *KLF3* in each cell type.

### 2.7 Association of *KLF3* expression with the tumor microenvironment (TME) and immune checkpoints

To evaluate the relationship between *KLF3* expression and TME, the stromal, immune, and ESTIMATE scores of each patient in each tumor were calculated according to the *KLF3* gene expression using the R package ESTIMATE (25). Further, Spearman’s correlation coefficient of *KLF3* expression and immune infiltration score in each tumor was calculated using the corr.test function of the R package psych (version 2.1.6).

Further, extract the expression data of 60 immune checkpoint pathway genes (26) (including Inhibitory (24) and Stimulatory (36)) in each sample, analyze the expression relationship between *KLF3* and immune checkpoint genes based on Spearman correlation, and use Heatmap for visualization. The TIMER2.0 database (http://timer.comp-genomics.org/) was used to analyze the correlation of target genes with marker genes related to T-cell exhaustion (27–29), M2 macrophages (30) and cancer-associated fibroblasts (CAFs) (31).

### 2.8 *KLF3* expression and immunotherapy response and drug prediction

As in previous studies (32), we calculated the Tumor mutation burden(TMB) of each tumor using the TMB function of the R package maftools (version 2.8.05) and obtained pan-cancer Microsatellite instability(MSI)data (33). The correlation between *KLF3* expression and TMB/MSI of each cancer type was calculated by the Spearman method, and visualized by radar map. Immunotherapy response prediction and biomarker assessment of *KLF3* were predicted from the TIDE website (http://tide.dfci.harvard.edu). Based on the GDSC and CTRP databases, the GSCA online website (http://bioinfo.life.hust.edu.cn/GSCA/#/drug) was used to predict the *KLF3* targeted sensitive drugs, and the bubble chart displays the relationship between the drug’s half-inhibitory concentration (IC50) and *KLF3* expression.

### 2.9 *In vitro* experiments

Cell culture, plasmid transfection, RNA extraction, quantitative real-time PCR, and immunoblotting were in agreement with previous studies (34). PANC-1 and SW1990 were purchased from the National Cell Identification and Collection Center of the Chinese Academy of Sciences. BxPC-3 (CL-0042) was purchased from Procell (Wuhan, China). The HPDE6-C7 cell line has been preserved by our laboratory. All cell lines in this experiment were identified and verified by short tandem repeat sequences. Cell culture dishes and 6-well plates were obtained from NEST Biotechnology (Wuxi, China). RNA duplexes were designed and synthesized by the Genepharma Company (Shanghai, China). **Table S1** lists the sequences of the shRNA and PCR primers used in this study. Primary antibodies were as follows: *KLF3* (Abcam, 1:500) and β-Tubulin (proteintech, 1:1000). Cell counting kit-8 (CCK-8), 5-Ethynyl-2’-Deoxyuridine (EdU), wound healing assay and transwell assay experimental details were consistent with previous studies (35). Immunocytochemistry and immunofluorescence (ICC/IF) were conducted as previously described (34). The corresponding antibodies are: *KLF3* Rabbit pAb(1:100, A7195, ABclonal) and Goat anti-Rabbit IgG (H+L) Cross-Adsorbed Secondary Antibody, Alexa Fluor™ 546 (1:1000, A-11080, Thermo Fisher).

### 2.10 Subcutaneous xenograft model

Female nude (BALB/c) mice (4 weeks old) were obtained from Hangzhou Ziyuan Experimental Animal Science and Technology Co., Ltd. After acclimatizing the BALB/c nude mice to the housing conditions for one week, they were randomly allocated into two groups: sh-NC and sh-*KLF3*#1. PANC-1 cells in the logarithmic growth phase, stably transfected with sh-NC and sh-*KLF3*#1, were harvested and suspended in PBS to achieve a cell density of 2×10^7^ cells/mL. The lower dorsal region of each nude mouse was disinfected, followed by the subcutaneous injection of 100 μL of cell suspension. Tumor volume was assessed every 5 days by the following formula: volume = length × width^2^ × 0.5. Mice were euthanized on day 35 after inoculation, and the tumors were removed and weighed. Animal experiments were approved by the Animal Experimental Ethical Inspection of Nanchang Royo Biotech Co. Ltd. (RYE2022092401).

### 2.11 Statistical analysis

All data were analyzed using GraphPad Prism 8.0 (GraphPad, San Diego, USA). The bioinformatics analysis in this study was partially supported by Sangerbox (http://vip.sangerbox.com/). To assess the significance of differences between the two groups, a Student’s t-test was conducted. Furthermore, paired t-tests were performed to compare the expression levels of *KLF3* in tumor tissues with those in their paired normal tissues. The Spearman correlation coefficient was used to evaluate associations between variables. The Log-rank test was used in survival analysis. For all statistical comparisons, significance levels were set at p < 0.05.

## 3 Results

### 3.1 Differential expression and localization of *KLF3*

**Figure 1:**
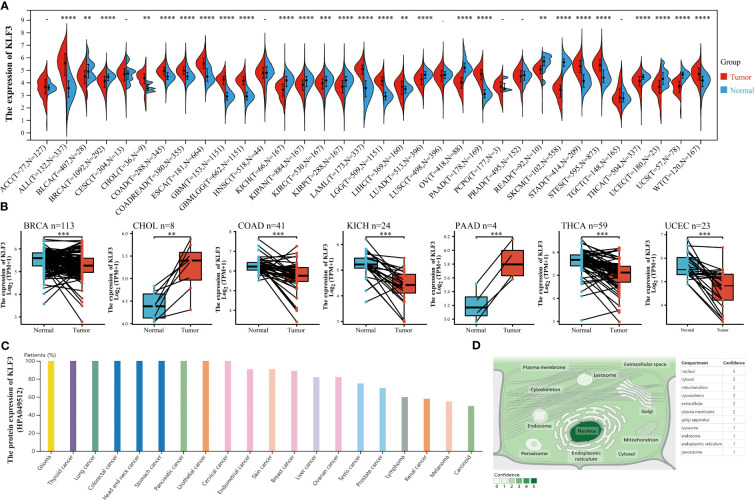
Differences in expression and localization of *KLF3* in pan-cancer. **(A)** *KLF3* mRNA expression levels in pan-cancer and normal tissues based on TCGA and GTEx databases. **(B)** Differential expression of *KLF3* in cancers and paired normal tissues were obtained through the TCGA database. **(C)** *KLF3* protein expression levels were obtained from the HPA database. **(D)** *KLF3* expression mapping was obtained from the Genecards database. **p < 0.01, ***p < 0.001, ****p < 0.0001.

In order to investigate the expression differences of *KLF3* in tumor and normal tissues in pan-cancer, we conducted the following analysis. By integrating the TGCA and GTEx databases, we found that *KLF3* mRNA was significantly upregulated in 14 types of tumors (ALL, CHOL, COAD, COADREAD, ESCA, GBM, GBMLGG, LAML, LGG, LIHC, PAAD, STAD, STES, and WT; all p<0.01), while it was significantly downregulated in 13 types of tumors (BLCA, BRCA, KICH, KIPAN, KIRC, KIRP, LUAD, OV, READ, SKCM, THCA, UCEC, and UCS; all p < 0.01), compared with normal tissues (**Figure 1A**). Furthermore, through analysis of paired cancer and normal tissues in the TCGA database, we found that *KLF3* mRNA was upregulated in CHOL (p < 0.01) and PAAD (p < 0.001), but significantly downregulated in BRCA (p < 0.001), COAD (p < 0.001), KICH (p < 0.001), THCA (p < 0.001), and UCEC (p < 0.001) compared with paired normal tissues (**Figure 1B**). These results demonstrate a consistent trend between paired and unpaired sample analyses, except for COAD. Further analysis through the Human Protein Atlas (HPA) database revealed that the expression frequency of *KLF3* protein was 100% in tumor types such as glioma (11/11), thyroid cancer (3/3), lung cancer (10/10), colorectal cancer (10/10), head and neck cancer (4/4), stomach cancer (12/12), urothelial cancer (12/12), cervical cancer (11/11), and pancreatic cancer (11/11), while the lowest expression frequency was observed in tumor types such as carcinoid (2/4), melanoma (6/11), and renal cancer (7/12) (**Figure 1C**). Moreover, utilizing the Genecards database, we observed that *KLF3* was mainly localized in the nucleus (**Figure 1D**).

### 3.2 Genetic changes and epigenetic modification of *KLF3*

**Figure 2:**
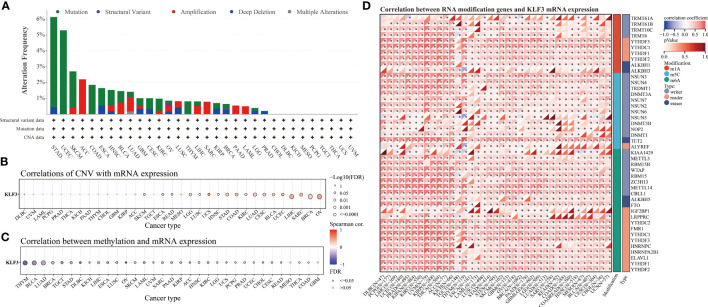
Genetic alteration and epigenetic modification of *KLF3*. **(A)** From the cBioPortal website, Mutation types and frequencies of *KLF3* in pan-cancer were identified. **(B, C)** In pan-cancer, the relationship between *KLF3* expression and gene copy number variation(CNV) and methylation. **(D)** Spearman correlation of *KLF3* expression with RNA-associated modification (m1A, m5C, m6A) gene expression. Blue to red within the triangle on the left side of the heatmap indicates a low to high correlation. In the bar graph on the right, red represents m1A-related genes, blue represents m5C-related genes, and green represents m6A-related genes. *p < 0.05.

Since differential expression of *KLF3* was observed in tumors, we analyzed its genetic alterations and epigenetic regulatory modifications using the online resources cBioPortal and GSCALite. As shown in **Figure 2A**, the main genetic alterations type of *KLF3* was “mutation”, among which STAD (5.68%), UCEC (5.29%), SKCM (2.25%), COAD (1.85%) and ESCA (1.1%) were the most typical. “Amplification” was mainly seen in ACC (2.2%), LUAD (0.88%), SARC (0.78%), BLAC (0.73%), and PAAD (0.54%). In pan-cancer, the frequency of *KLF3* gene mutations in “deep deletion”, “structural variation” and “multiple Alterations” was generally less than 0.5%. CNVs are important aberrations that result in alterations in gene expression in tumorigenesis and tumor growth (36). Spearman correlation analysis showed that the mRNA expression of *KLF3* was significantly positively correlated with the CNV of the *KLF3* gene in SKCM, ESCA, READ, LGG, LUSC, UCS, HNSC, STAD, COAD, KIRC, LUAD, CESC, BLCA, UCEC, LIHC, SARC, BRCA, and OV (**Figure 2B** and **Table 2**). Dysregulation of DNA methylation is strongly associated with the onset of various diseases including cancer (37). The GSCA database provided the methylation sites most negatively correlated with *KLF3* gene expression in each tumor type (**Figure 2C** and **Table 3**). Further through the UALCAN database, we found that the methylation level of the *KLF3* gene promoter in BLCA, BRCA, CESC, ESCA, HNSC, KIRC, LUAD, LUSC, PRAD, TGCT, and UCEC was significantly higher than that in corresponding normal tissues; The opposite phenomenon occurs in STAD and THCA (**Figures S2A–W**). Accumulating evidence suggests that RNA modification pathways were misregulated in human cancers and may be ideal targets for cancer therapy (38). The association between *KLF3* expression and RNA modification-related genes was shown in **Figure 2D**. We found that *KLF3* expression was generally positively correlated with m1A, m5C, and m6A-related gene expression in pan-cancer, especially in YTHDF1, NSUN3, TET2, METTL14, YTHDC2, and FMR1. The above results indicate that the abnormal expression of *KLF3* in different tumors may be closely related to its gene variation and participation in epigenetic modification.

**Table 2:** Summary of correlations between *KLF3* mRNA expression and gene copy number variation (CNV) across tumor types.

Cancer type	Gene symbol	Spearman correlation	False discovery rate
ACC	*KLF3*	0.269568086	0.080558731
BLCA	*KLF3*	0.213903363	0.000060285
BRCA	*KLF3*	0.250443714	3.57882E-16
CESC	*KLF3*	0.276939696	9.76082E-06
CHOL	*KLF3*	0.235854534	0.616429399
COAD	*KLF3*	0.285902934	7.87733E-06
DLBC	*KLF3*	-0.463891707	0.5319788
ESCA	*KLF3*	0.218895292	0.009720158
GBM	*KLF3*	-0.050440564	0.811144689
HNSC	*KLF3*	0.111390536	0.026646157
KICH	*KLF3*	0.064661608	0.891817142
KIRC	*KLF3*	0.151791374	0.00302508
KIRP	*KLF3*	0.066959676	0.465886185
LAML	*KLF3*	0.057608731	0.999933068
LGG	*KLF3*	0.204484747	0.000033171
LIHC	*KLF3*	0.402389368	4.95043E-14
LUAD	*KLF3*	0.267243738	4.27216E-09
LUSC	*KLF3*	0.10793903	0.029197237
MESO	*KLF3*	0.284482237	0.063975694
OV	*KLF3*	0.456654684	4.46299E-16
PAAD	*KLF3*	0.048937431	0.722535439
PCPG	*KLF3*	-0.048820375	0.842969495
PRAD	*KLF3*	0.028446219	0.790786918
READ	*KLF3*	0.282768295	0.031129082
SARC	*KLF3*	0.337299222	3.03286E-07
SKCM	*KLF3*	0.161511884	0.00598773
STAD	*KLF3*	0.254859208	1.01316E-06
TGCT	*KLF3*	0.073228188	0.59569208
THCA	*KLF3*	0.052035206	0.867102507
THYM	*KLF3*	0.13299963	0.639610588
UCEC	*KLF3*	0.343682486	0.000042067
UCS	*KLF3*	0.493181122	0.001726227
UVM	*KLF3*	0.135860527	0.805378483

**Table 3:** Summary of methylation sites most negatively associated with *KLF3* gene expression by tumor type.

Cancer type	Gene symbol	Tag	Spearman correlation	False discovery rate
ACC	*KLF3*	cg14848077_*KLF3*	-0.36436222	0.001035081
BLCA	*KLF3*	cg05205842_*KLF3*	-0.497195632	0
BRCA	*KLF3*	cg05205842_*KLF3*	-0.299476536	2.37906E-15
CESC	*KLF3*	cg05205842_*KLF3*	-0.180134377	0.001636688
CHOL	*KLF3*	cg05205842_*KLF3*	-0.382496782	0.021963799
COAD	*KLF3*	cg24491704_*KLF3*	-0.121730979	0.045723365
DLBC	*KLF3*	cg05562080_*KLF3*	-0.610942249	6.45225E-06
ESCA	*KLF3*	cg05205842_*KLF3*	-0.367565641	3.49392E-07
GBM	*KLF3*	cg22453435_*KLF3*	-0.203348416	0.15203925
HNSC	*KLF3*	cg05205842_*KLF3*	-0.186902774	0.000018519
KICH	*KLF3*	cg14848077_*KLF3*	-0.28713078	0.019719099
KIRC	*KLF3*	cg22051776_*KLF3*	-0.249715879	7.03871E-06
KIRP	*KLF3*	cg05205842_*KLF3*	-0.278751613	3.05599E-06
LAML	*KLF3*	cg22453435_*KLF3*	-0.363098662	1.35426E-06
LGG	*KLF3*	cg22453435_*KLF3*	-0.152846655	0.000500724
LIHC	*KLF3*	cg22453435_*KLF3*	-0.292211488	1.14367E-08
LUAD	*KLF3*	cg24279243_*KLF3*	-0.386952517	0
LUSC	*KLF3*	cg05205842_*KLF3*	-0.268445047	1.7018E-07
MESO	*KLF3*	cg03910048_*KLF3*	-0.19820296	0.065831082
OV	*KLF3*	cg17074863_*KLF3*	-0.833333333	0.008267196
PAAD	*KLF3*	cg05205842_*KLF3*	-0.324595794	0.000011025
PCPG	*KLF3*	cg09915299_*KLF3*	-0.170221581	0.022824428
PRAD	*KLF3*	cg21953508_*KLF3*	-0.166916775	0.000189147
READ	*KLF3*	cg22051776_*KLF3*	-0.237211596	0.023014261
SARC	*KLF3*	cg22453435_*KLF3*	-0.275770992	7.24367E-06
SKCM	*KLF3*	cg22453435_*KLF3*	-0.210415656	4.52597E-06
STAD	*KLF3*	cg05205842_*KLF3*	-0.319469364	3.58383E-10
TGCT	*KLF3*	cg14848077_*KLF3*	-0.497559892	1.17E-10
THCA	*KLF3*	cg14848077_*KLF3*	-0.113606969	0.010620333
THYM	*KLF3*	cg05205842_*KLF3*	-0.678359608	0
UCEC	*KLF3*	cg24491704_*KLF3*	-0.182558493	0.016639949
UCS	*KLF3*	cg05205842_*KLF3*	-0.32635468	0.013572273
UVM	*KLF3*	cg18361445_*KLF3*	-0.274566338	0.013949947

### 3.3 Correlation between *KLF3* expression and clinicopathological features and prognosis

**Figure 3:**
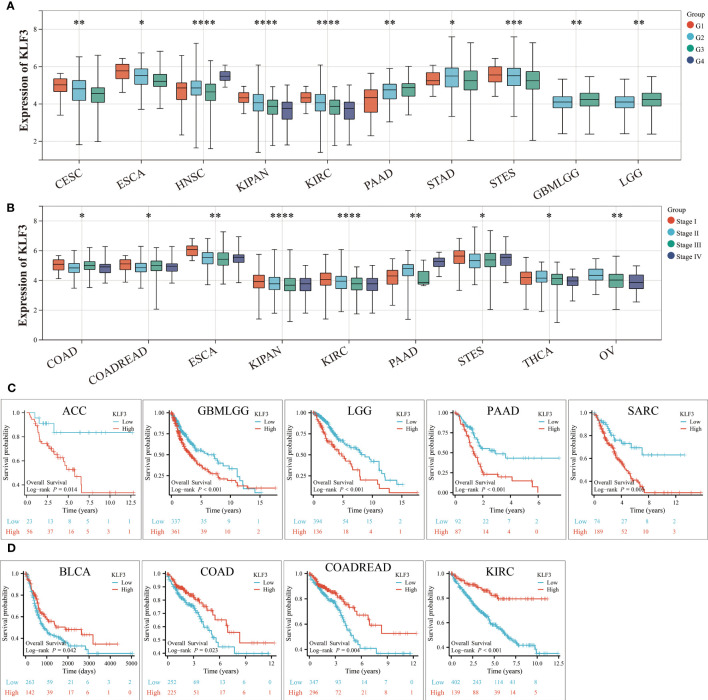
Relationship between *KLF3* expression and clinical characteristics and prognosis. **(A)** Relationship of *KLF3* expression to tumor histological grading. **(B)** Relationship between *KLF3* expression and tumor clinical staging. **(C)** Survival differences of *KLF3* high and low expression groups in ACC, GBMLGG, LGG, PAAD, and SARC. **(D)** Survival differences of *KLF3* high and low expression groups in BLCA, COAD, COADREAD, and KIRC. The Log-rank method was used to compare the difference in survival between the high-expression group and the low-expression group. Only cancer species with statistically significant differences were shown. *p < 0.05, **p < 0.01, ***p < 0.001, ****p < 0.0001.

The above results indicate that *KLF3* was abnormally expressed in a variety of tumors, but whether its expression is related to tumor progression needs further exploration. According to the results shown in **Figure 3A**, it was observed that as the histological grades increased in patients with CESC, ESCA, KIPAN, KIRC, and STES, there was a decreasing trend in *KLF3* expression. Conversely, the opposite trend was observed in patients with PAAD, HNSC, GBMLGG, and LGG (all p<0.05). Furthermore, it was also observed that as the clinical stages progressed in patients with COAD, COADREAD, ESCA, KIPAN, KIRC, THCA, and OV, there was a decreasing trend in *KLF3* expression, except for PAAD patients where the opposite trend was observed (**Figure 3B**, all p<0.05). Next, by drawing the Kaplan-Meier survival curve, we found that compared with patients in the *KLF3* low expression group, high *KLF3* expression was closely related to shorter overall survival in patients with ACC, GBMLGG, LGG, PAAD, and SARC (all p<0.05, **Figure 3C**). In contrast, high expression of *KLF3* was closely associated with good prognosis in patients with BLCA, COADREAD, COAD, and KIRC (all p<0.05, **Figure 3D**). Further, we established a COX proportional regression model on the pan-cancer patient survival data and *KLF3* expression to analyze the relationship between *KLF3* gene expression and prognosis in each tumor. The results showed that higher *KLF3* expression was associated with poorer OS in LGG, GBMLGG, ACC, and PAAD, whereas the opposite results were observed in patients with KIRC, COADREAD, COAD, and KIPAN (**Figure S2A**). DSS results showed that higher *KLF3* expression was associated with poorer DSS in LGG, GBMLGG, PAAD, and ACC, whereas the opposite results were observed in patients with KIRC and KIPAN (**Figure S2B**). **Figure S2C** shows that high *KLF3* expression was associated with poorer PFI in ACC, LGG, GBMLGG, UVM, and PAAD, whereas better in KIRC, KIPAN, and HNSC. Furthermore, the expression level of *KLF3* was positively correlated with poorer DFI in PAAD and ACC (**Figure S2D**). Taken together, the results suggest that *KLF3* can effectively predict the prognosis of multiple cancers, most notably in PAAD.

### 3.4 The function analysis of *KLF3* in pan-cancer

**Figure 4:**
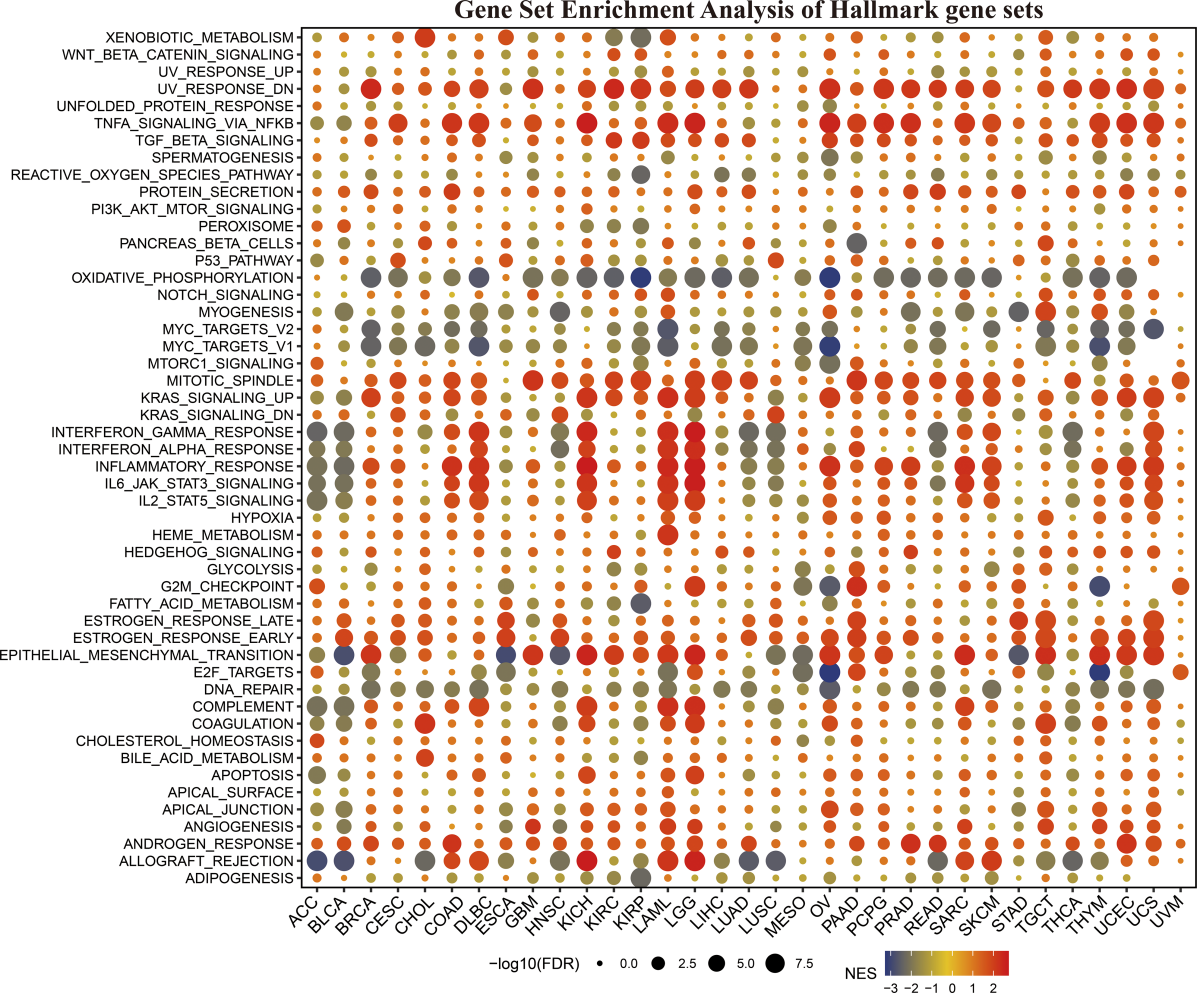
The hallmarks gene set enrichment analysis (GSEA) of *KLF3* in various types of cancer. FDR, Error discovery rate. NES, Standardized enrichment fraction.

To clarify how *KLF3* affects prognosis, we analyzed the correlation between *KLF3* and 14 functional states using single-cell sequence data from CancerSEA. As shown in **Figure S3**, *KLF3* expression was negatively correlated with the cell cycle, DNA damage cancer injury, DNA repair, and invasive ability of most tumors, while positively correlated with tumor differentiation, EMT, hypoxia, inflammation, metastasis, proliferation, quiescence, and stemness. In addition, through GSEA, we explored the possible signaling pathways through which the abnormal expression of *KLF3* affects the above functions (**Figure 4**). We found significant enrichment of immune-related signaling pathways in most tumor types, including TNFA-signaling-via-NFκB, IFN- γ response, IFN- α response, inflammatory response, IL6-JAK-STAT3, IL2-STAT5, and allograft-rejection. The results also showed that various tumor types were enriched in TGF-β, protein slicing, oxidative phosphorylation, mTORC1, KRAS, epithelial-mesenchymal transition, and DNA repair signals. The above results indicate that *KLF3* is closely related to tumor progression and immune response.

### 3.5 Relationship between *KLF3* expression and TME

**Figure 5:**
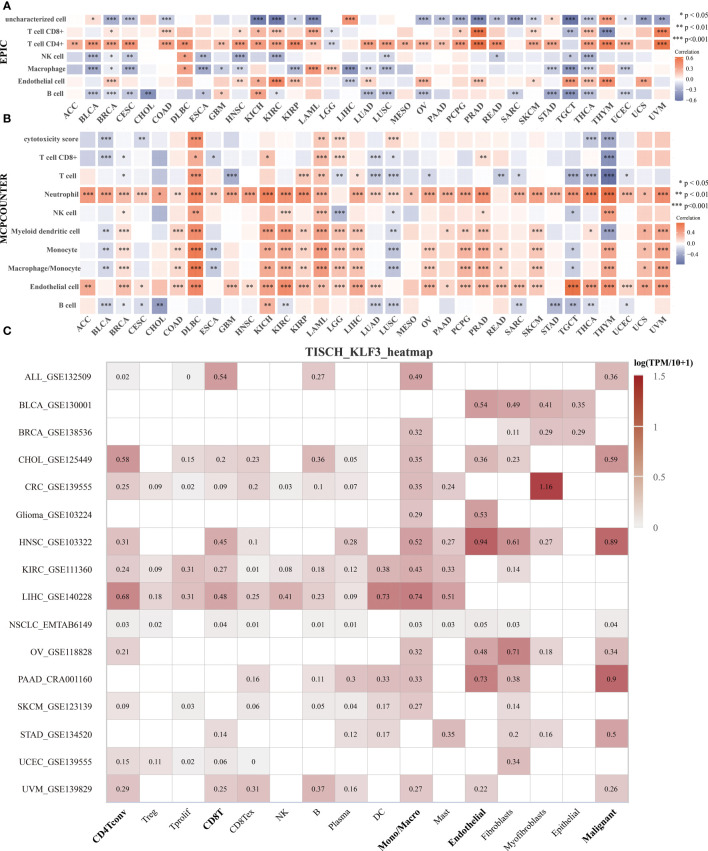
Expression and distribution of *KLF3* in the TME. **(A, B)** Analysis of immune cell infiltration status by EPIC **(A)** and MCP-counter **(B)** algorithm. **(C)** The expression of *KLF3* in different cell types was analyzed by the TISCH2 website. *p < 0.05, **p < 0.01, ***p < 0.001.

**Figure 6:**
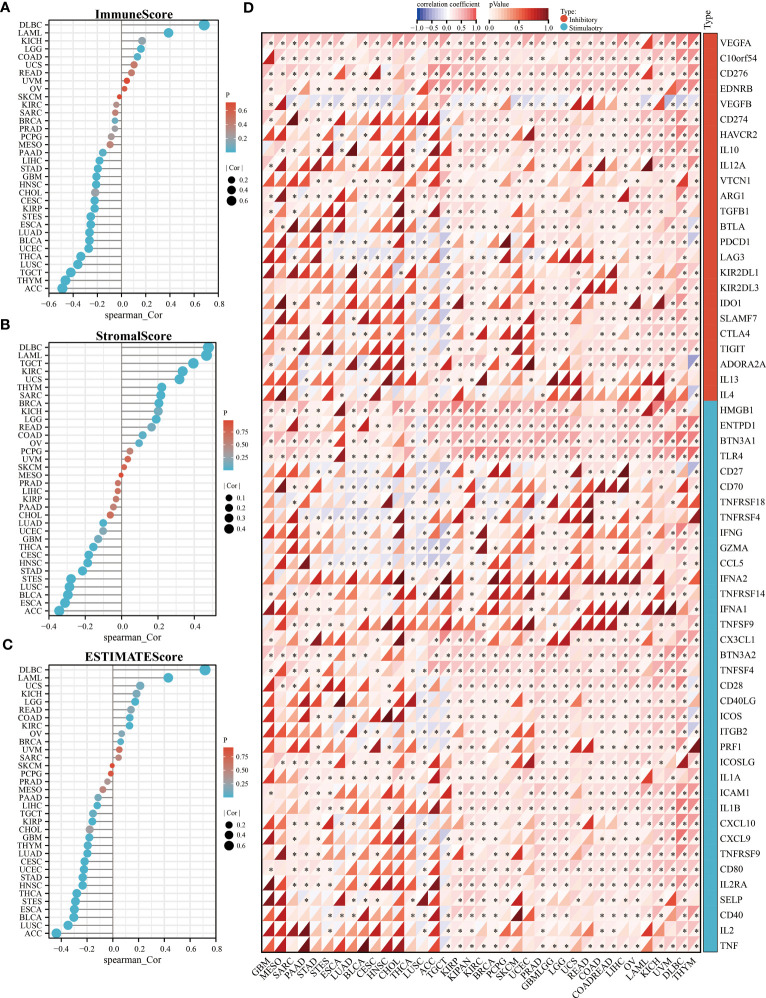
Relationship of *KLF3* expression with TME in pan-cancer. **(A-C)** Correlation of *KLF3* expression with immune score **(A)**, stromal score **(B)**, and estimated score **(C)** in pan-cancer. **(D)** Analysis of expression correlation of *KLF3* expression with immune inhibitory and stimulatory genes. *p < 0.05.

To clarify the relationship between *KLF3* and immune cell infiltration, we analyzed it by EPIC and MCPCOUNTER algorithms. The results showed that the expression of *KLF3* was closely related to the infiltration of CD4+ T cells, CD8+ T cells, neutrophils, myeloid dendritic cells, monocytes/macrophages, and endothelial cells in most of the TME (**Figures 5A, B**). We further verified the above findings by analyzing single-cell sequencing data. As shown in **Figure 5C**, *KLF3* was expressed in higher proportions in CD4Tconv, CD8T, monocytes/macrophages, endothelial cells, and malignant cells of the TME. Then we analyzed the relationship between *KLF3* expression and TME in pan-cancer. *KLF3* expression was negatively correlated with the immune scores of ACC, THYM, TGCT, LUSC, THCA, UCEC, BLCA, LUAD, ESCA, STES, KIRP, CESC, CHOL, HNSC, GBM, STAD, LIHC, and PAAD; and positively correlated with the immune scores of COAD, LGG, LAML, and DLBC (**Figure 6A**). *KLF3* expression was negatively correlated with stromal scores for ACC, ESCA, BLCA, LUSC, STES, STAD, HNSC, CESC, THCA, GBM, UCEC, and LUAD, and positively correlated with LGG, KICH, BRCA, SARC, THYM, UCS, KIRC, TGCT, LAML and DLBC (**Figure 6B**). *KLF3* expression was negatively correlated with estimated scores for ACC, LUSC, BLCA, ESCA, STES, THCA, HNSC, STAD, UCEC, CESC, LUAD, THYM, GBM, CHOL, KIRP, TGCT, LIHC, and PAAD, and positively correlated with the estimated scores of KIRC, COAD, READ, LGG, KICH, USC, LAML, DLBC, OV, and BRCA (**Figure 6C**). Spearman’s correlation analysis also showed that *KLF3* expression was significantly correlated with immune-related genes (**Figure 6D**). From a pan-cancer perspective, it was found that: immune-related genes *VEGFA, C10orf54, CD276, EDNRB, ARG1, HMGB1, ENTPD1, BTN3A1, TLR4, BTN3A2* were significantly positively correlated with *KLF3* expression, whereas *VEGFB* expression was negatively correlated with *KLF3* expression. Furthermore, we found that *KLF3* expression in BLCA was inversely correlated with the expression of related markers of T-cell exhaustion, M2 macrophages, and CAFs (**Figure S4**). Interestingly, we observed the opposite result in DLBC. From a pan-cancer perspective, *KLF3* was generally positively correlated with the expression of markers associated with M2 macrophages and CAFs. In addition, *KLF3* was mostly positively correlated with the expression levels of *TIGIT* among T-cell exhaustion genes(**Figure S4**). In short, the contribution of abnormal *KLF3* expression to TME is not negligible.

### 3.6 Predicting *KLF3*-related tumor immunotherapy responses and drugs

**Figure 7:**
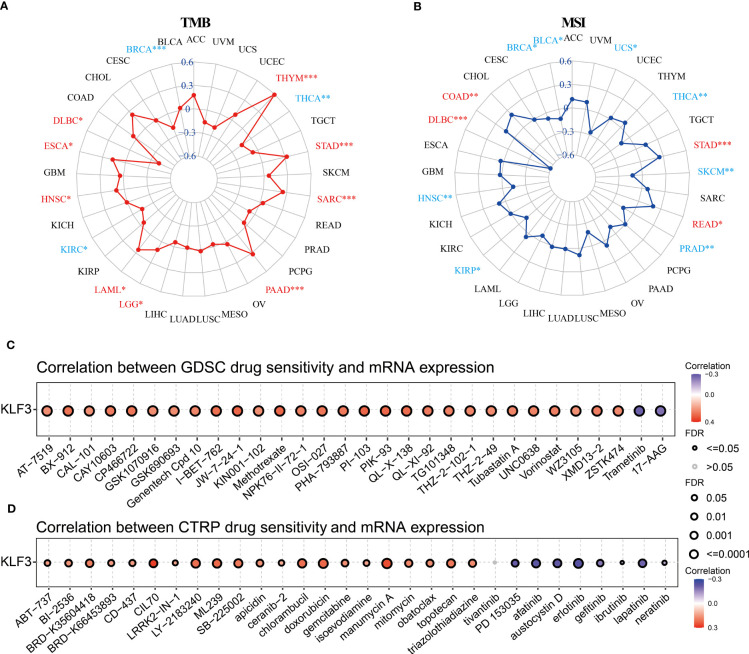
Immunotherapy response, biomarker correlation, and drug-sensitive prediction of *KLF3* in pan-cancer. **(A)** Radar chart showing the relationship between *KLF3* expression and TMB. **(B)** Radar chart showing the relationship between *KLF3* expression and MSI. GDSC **(C)** and CTRP **(D)** databases were used to predict the related drugs targeting *KLF3*. *p < 0.05, **p < 0.01, ***p < 0.001.

TMB and MSI are predictors of immunotherapy (39). Therefore, we evaluated the relationship between *KLF3* expression and TMB and MSI. Spearman’s correlation analysis showed that *KLF3* expression in DLBC(p<0.05), ESCA(p<0.05), HNSC(p<0.05), LAML(p<0.05), LGG(p<0.05), PAAD(p<0.001), SARC(p<0.001), STAD(p<0.001), and THYM(p<0.001) was positively correlated with their TMB, while it was negatively correlated with TMB values in BRCA(p<0.001), KIRC(p<0.05), THCA(p<0.01) (**Figure 7A**). In addition, *KLF3* expression levels were positively correlated with MSI in COAD(p<0.01), DLBC(p<0.001), READ(p<0.05), and STAD(p<0.001). In contrast, it was negatively correlated with MSI in BLCA(p<0.05), BRCA(p<0.05), HNSC(p<0.01), KIRP(p<0.05), PRAD(p<0.01), SKCM(p<0.01), THCA(p<0.01), and UCS(p<0.05) (**Figure 7B**). Further, we predicted the response and sensitivity of tumor patients to immunotherapy drugs based on *KLF3* expression. As shown in **Figure S5**, there were five mouse immunotherapy cohorts for which immunotherapy response could be predicted by *KLF3*. Notably, when comparing *KLF3* with common standard biomarkers of immunotherapy response, we found that an AUC greater than 0.5 was observed in 10 immunotherapy cohorts when *KLF3* alone was used as a predictive marker, indicating that *KLF3* outperformed TMB, T. Clonality, and B. Clonality in prediction (**Figure S6**). Subsequently, drug IC50 analysis of *KLF3* by the GDSC dataset revealed that trametinib (reversible inhibitor of mitogen-activated extracellular signal-regulated kinase 1 (MEK 1/2)), PD-0325901 (selective MEK inhibitor) and 17-AAG (HSP90 inhibitor) were the top three drugs negatively associated with *KLF3* expression; whereas PI-103 (multi-target PI3K inhibitor), JW-7-24-1 (small molecule kinase inhibitor) and PIK-93 (PI4KIIIβ inhibitor) were the top three drugs positively correlated with *KLF3* expression (**Figure 7C** and **Table S2**). Correlation of *KLF3* expression with drug IC50 based on the CTRP database showed that abiraterone (a CYP17 inhibitor), erlotinib (a tyrosine kinase inhibitor), and PD318088 (a non-ATP-competitive, MEK1/2-mutagenesis inhibitor) were the top three drugs negatively correlated with *KLF3* expression; manumycin A (a selective, competitive farnesyltransferase (FTase) inhibitor), CCT036477 (Wnt Pathway Inhibitor XI) and CIL70 were the top three drugs positively associated with *KLF3* expression (**Figure 7D** and **Table S3**). These results suggest a role for *KLF3* in predicting immunotherapeutic response in pan-cancer and predicting effective small molecule drugs targeting *KLF3*, which may provide strong evidence for future pan-cancer therapeutic studies.

### 3.7 Identification of *KLF3* in PAAD

**Figure 8:**
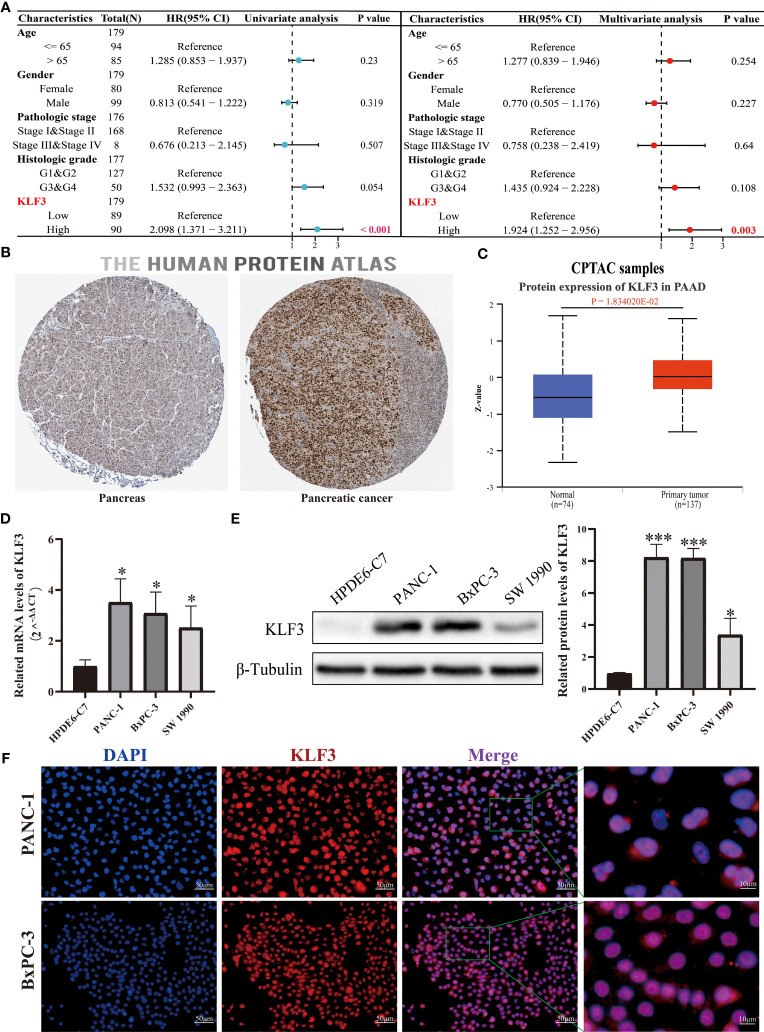
*KLF3* high expression was associated with a poor prognosis of PAAD. **(A)** The role of *KLF3* in PAAD was analyzed by univariate and multivariate Cox regression analysis. The data comes from the TCGA database. **(B)** Immunohistochemical staining was used to identify the expression of *KLF3* in PAAD and pancreatic tissues. **(C)** The CPTAC database was used to analyze the protein expression level of *KLF3* in PAAD and normal pancreas tissues. **(D, E)** The qPCR and Western blot were used to evaluate the basal expression levels of *KLF3* mRNA and protein in HDPE6-C7, PANC-1, BxPC-3, and SW 1990 cells. **(F)** ICC/IF was used to identify *KLF3* expression localization in PANC-1 and BxPC-3 cells. *p < 0.05, ***p < 0.001.

Through our analysis of *KLF3* in pan-cancer, we found that *KLF3* is significantly upregulated in PAAD (**Figures 1A–C**) and its expression positively correlates with patient clinical stage and histological grade (**Figures 3A, B**). It is also significantly associated with poor OS, DSS, PFI, and DFI of patients with PAAD (**Figure 3C** and **Figure S2**). Therefore, our study focused on investigating the oncogenic effect of *KLF3* in PAAD. First, we analyzed the clinical significance of *KLF3* in PAAD and its protein expression. The combined univariate and multifactorial COX regression analysis suggested that *KLF3* was an independent prognostic risk factor for PAAD (**Figure 8A**, all p<0.05). Subsequently, we characterized the protein expression of *KLF3* to clarify whether its mRNA expression was consistent with protein expression. The HPA database (40) showed that the intensity of immunohistochemical staining for *KLF3* was significantly higher in PAAD tissues than in normal pancreatic tissues (**Figure 8B**). This was validated by protein expression assay data from the CPTAC database (**Figure 8C**, p=0.01834020). The mRNA and protein basal expression levels of *KLF3* in normal pancreatic ductal epithelial cells and PAAD cell lines were detected using qPCR and western bot, respectively. As shown in **Figures 8D, E**, both mRNA and protein levels of *KLF3* were higher in PAAD cells than in normal pancreatic ductal epithelial cells HPDE6-C7(all p<0.05). The basal expression levels of *KLF3* were significantly higher in pancreatic cancer cell lines PANC-1 and BxPC-3 cells, which will serve as a tool cell for silencing *KLF3* expression. Based on ICC/IF analysis, it was found that *KLF3* expression was predominantly localized in the nucleus of PAAD cells (**Figure 8F**), which is in agreement with the information retrieved from the HPA (**Figure 8B**) and Genecards (**Figure 1D**) databases.

### 3.8 Silencing of *KLF3* inhibits PAAD progression

**Figure 9:**
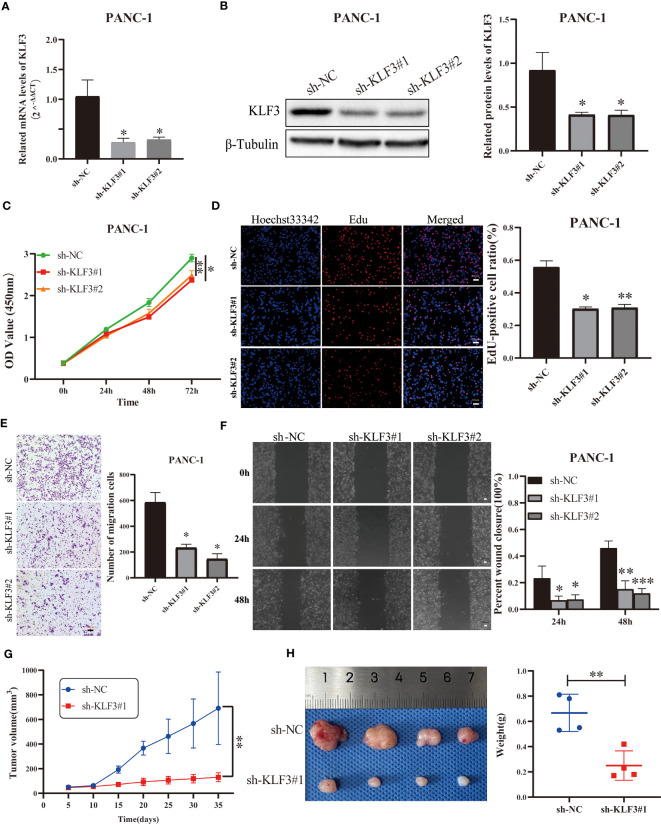
Silencing *KLF3* expression inhibits the progression of PAAD *in vivo* and *in vitro*. **(A, B)** qPCR and Western blot were used to detect the changes of *KLF3* mRNA and protein expression in PANC-1 cells transfected with sh-*KLF3* plasmid. **(C)** CCK-8 method was used to detect the changes in the viability of PANC-1 cells after transfection of sh-NC, sh-*KLF3*#1, and sh-*KLF3*#2 plasmids. **(D)** EdU staining was used to evaluate the changes in the proliferation ability of PANC-1 cells in sh-NC, sh-*KLF3*#1, and sh-*KLF3*#2 groups. Transwell assay **(E)** and wound healing assay **(F)** were used to evaluate the changes in the cell migration ability of PANC-1 cells in sh-NC, sh-*KLF3*#1, and sh-*KLF3*#2 groups. **(G, H)** The effect of *KLF3* silencing on the growth of subcutaneous xenograft tumors(n=4), presented as tumor growth volume curves **(G)**, dissected tumor representative graphs, and weight graphs **(H)**, respectively. *p < 0.05, **p < 0.01, ***p < 0.001.

We effectively inhibited the expression of *KLF3* using RNAi technology (**Figures 9A, B**, **S7A, B**, all p<0.05). By CCK-8 assay we found that interfering with *KLF3* expression would inhibit cell viability of PANC-1 and BxPC-3 cells (**Figures 9C**, **S7C**, all p<0.05). Similarly, we used EdU cell proliferation assays to find that the knockdown of *KLF3* expression would inhibit the proliferative capacity of PAAD cell lines (**Figures 9D**, **S7D**, all p<0.05). Subsequently, Transwell and wound healing assays were used to examine the potential role of *KLF3* in the migration of PAAD and BxPC-3 cells. As shown in **Figures 9E, F**, **S7E, F**, the cell migration ability of PANC-1 and BxPC-3 cells with disrupted *KLF3* expression were significantly inhibited(all p<0.05). Further, *in vivo* experiments revealed that tumor growth was significantly slower in PANC-1 cells with stably silenced *KLF3* expression compared to the control group (**Figure 9G**, p<0.01). The weight of tumors in the sh-*KLF3*#1 group was also significantly reduced at the termination of the experiment (**Figure 9H**, p<0.01). Both *in vitro* and *in vivo* experiments indicated that *KLF3* is a risk factor for PAAD and that silencing *KLF3* expression would inhibit the progression of PAAD.

## 4 Discussion

Krüppel-like factors (KLF) are a family of eukaryotic DNA-binding transcriptional regulators involved in a variety of essential cellular functions, including proliferation, differentiation, migration, inflammation, and pluripotency (41). The common feature of most of its members is that their binding sites are not the same in different cells and environments: they may also bind different sites in the same cell and control different genomes in response to different microenvironments (41). *KLF3*, a member of the KLF, binds cofactor C-terminal binding proteins, which in turn recruit a large repressor complex to mediate transcriptional silencing (8). In recent years, studies on *KLF3* have focused on the regulation of the production of erythroid (42), B-cell (43), lymphocyte (44), and adipose (45) substances, while few reports have explored its role in tumors. In this study, a systematic analysis of *KLF3* expression profile, genetic alteration, DNA methylation, RNA modification, clinical significance, and prognostic value in pan-cancer was performed. Further correlations between *KLF3* expression and TME, immune cell infiltration, immune checkpoints, immunotherapeutic response, and small molecule drug prediction were analyzed. This study also clarified the oncogenic role of *KLF3* in PAAD through functional experiments.

It has been shown that *KLF3* was aberrantly expressed in tumors and correlates with prognosis. For example, Huang et al. reported that *KLF3* was lowly expressed in colorectal cancer and associated with poor prognosis (10). Shan et al. demonstrated that *KLF3* was highly expressed in osteosarcoma and associated with poor prognosis (46). Wei et al. demonstrated that *KLF3* was lowly expressed in lung cancer and associated with poor prognosis (14). In contrast, Meng et al. showed that *KLF3* was lowly expressed in prostate cancer and was associated with favorable recurrence-free survival time (47). Our study also found that *KLF3* mRNA was significantly upregulated in 14 tumor types and significantly downregulated in 13 tumor types compared to normal tissue. In this study, we found that the abnormal expression of *KLF3* is affected by many factors, and its abnormal expression cannot be directly explained by genetic alteration, CNV, and methylation modification. Its abnormal expression is also regulated by other mechanisms, which requires more precise exploration in the future. In addition, increased *KLF3* expression was negatively associated with histological grade in CESC, ESCA, KIPAN, KIRC, and STES and positively associated with histological grade in PAAD, HNSC, GBMLGG, and LGG. *KLF3* expression was also negatively associated with clinical stage progression in COAD, COADREAD, ESCA, KIPAN, KIRC, THCA, and OV and positively associated with a clinical stage in PAAD. Further survival analysis revealed that high *KLF3* expression was strongly associated with poor prognosis in patients with ACC, GBMLGG, LGG, PAAD, and SARC. In contrast, it was associated with a good prognosis in patients with BLCA, COADREAD, COAD, and KIRC, which is consistent with previous findings in colorectal cancer (10, 48). Our results also further validate that KLFs family genes were differentially expressed in different tumors or settings (41). Through the above analysis, we found a prominent role for *KLF3* in PAAD. Previous studies have shown that miR-324-5p promotes pancreatic cancer cell proliferation and apoptosis by targeting *KLF3* (13). However, in this study, *KLF3* was found to be highly expressed in PAAD at both mRNA and protein levels. Increased *KLF3* expression was strongly associated with histological grade, clinical stage, and poor prognosis (OS/DSS/PFI/DFI) of PAAD. Univariate and multifactorial Cox regression analyses identified *KLF3* as an independent prognostic risk factor for PAAD. *In vitro* and *in vivo* experiments also found that inhibition of *KLF3* expression would inhibit the proliferation and migratory capacity of PAAD cells. Further, single-cell sequencing data also revealed that *KLF3* expression was positively correlated with EMT, hypoxia, inflammation, metastasis, and proliferation in most tumors, which was further validation of our functional assay results. Regarding the specific mechanism of *KLF3* abnormal expression in promoting or inhibiting tumors, we found that it is mainly enriched in TGF-β, oxidative phosphorylation, mTORC1, KRAS, and EMT signaling pathways through GSEA. Previous studies have also found that downregulation of *KLF3* expression inhibits the progression of lung cancer by inhibiting the JAK2/STAT3 and PI3K/AKT signaling pathways (49); *KLF3* silencing promotes lung cancer EMT and enhances lung cancer metastasis through the *STAT3* signaling pathway (14); *KLF3* activates WNT through WNT1/β-catenin signaling pathway to promote the growth and metastasis of gastric cancer (50); of course, the specific mechanism of *KLF3* regulating tumors is not comprehensive enough, and more in-depth mechanism exploration is needed in the future. In summary, *KLF3* can effectively predict the prognosis of many cancers and is most evident in PAAD.

There is growing interest in the significance of TME in tumor progression, prognosis, and therapeutic responsiveness. Immune cells within the TME can promote or suppress tumor growth (51). Previous studies suggested that *KLF3* may interact with *KLF2* in controlling the differentiation/homeostasis of certain B-cell subpopulations (52). For example, B-cell development was impaired in the absence of *KLF3* (43), while *KLF3* overexpression resulted in a significant increase in the number of B-cells in the marginal zone of the spleen (53). In addition, *KLF3* directly inhibited transcription of the inflammatory regulator Galectin-3; *KLF3* suppressed NF-κB-driven inflammation in mice (54); and eosinophil function was also regulated in adipose tissue (55). However, the relationship between *KLF3* and pan-cancer TME and tumor immune cell infiltration remains largely unknown. In this study, we found that *KLF3* expression was negatively associated with immune scores in the TME of most tumors. EPIC and MCPCOUNTER algorithm analysis revealed that *KLF3* expression was strongly associated with CD4+ T cells, CD8+ T cells, neutrophils, myeloid dendritic cells, monocytes/macrophages, and endothelial cell infiltration in TME. We also validated this result by analysis of single-cell sequencing data. This study found that the expression of *KLF3* was roughly positively correlated with the expression of genes related to M2 macrophages and CAFs, which may suggest that the high expression of *KLF3* can promote the formation of a microenvironment suitable for tumor cell growth. Previous studies have also suggested that *KLF4*, also a member of the KLF family, can regulate the polarization of M1/M2 macrophages in alcoholic liver disease (31). In conclusion, *KLF3* expression is closely associated with the composition of TME.

In advanced cancers, immunotherapy is effective in multiple clinical trials (56), but only a small number of patients can benefit from it (57). Therefore, the development of biomarkers that effectively predict response to immunotherapy is essential to screen potential populations that may benefit from immunotherapy. *PD-L1* expression and genomic features (e.g. oncogenic driver mutations, TMB and MSI) have been proposed as biomarkers of response to immunotherapy (58). In this study, *KLF3* expression was correlated with pan-cancer TMB, MSI, immune activation/inhibition-related genes, T-cell exhaustion, M2 macrophages and CAFs-related genes, and TME. To validate the value of *KLF3* in predicting response to immunotherapy, we also calculated its ROC value as a biomarker in the immunotherapy cohort. Interestingly, the immunotherapeutic response was predicted by *KLF3* in five mouse immunotherapy cohorts; and *KLF3* outperformed TMB, T. Clonality, and B. Clonality when used alone as a predictive marker. However, there is no evidence to support whether *KLF3* can be used as a tumor cell signaling protein for CAR-T therapy, which requires further exploration in the future. Finally, we also predicted a series of small molecules targeting *KLF3* through the GDSC and CTRP databases, which will provide a basis for the future development of immunotherapeutic and targeted therapeutic agents.

## 5 Conclusion

In this study, *KLF3* was aberrantly expressed in a variety of tumor types and was strongly correlated with clinical progression and prognosis; *KLF3* could be a potential prognostic marker, especially in PAAD. In addition, the contribution of *KLF3* to TME and the abundance of immune cell infiltration is not negligible. It may be a biomarker for predicting response to immunotherapy and has the potential to guide individualized immunotherapy for cancer.

## Data availability statement

Publicly available datasets were analyzed in this study. This data can be found here: the TCGA and GTEx databases were downloaded from the UCSC (https://xenabrowser.net/) database; the Human Protein Atlas (HPA, https://www.proteinatlas.org/); TISCH2, http://tisch.comp-genomics.org/home/, select datasets: ALL-GSE132509, BLCA-GSE130001, BRCA-GSE138536, CHOL-GSE125449, CRC-GSE139555, Glioma-GSE103224, HNSC-GSE103322, KIRC-GSE111360, LIHC-GSE140228, NSCLC-EMTAB6149, OV-GSE118828, PAAD-CRA001160, SKCM-GSE123139, STAD-GSE134520, UCEC-GSE139555 and UVM-GSE139829.

## Ethics statement

The animal study was reviewed and approved by Animal Experimental Ethical Inspection of Nanchang Royo Biotech Co. Ltd. (RYE2022092401).

## Author contributions

JZ and HT conducted the formal analysis and wrote the original draft; YZ performed the project administration; HT conducted the experiments. JZ, XZ, JY, and QZ participated in software analysis; JZ, XZ, HT, and JY conducted data curation; JZ and YZ contributed to writing, reviewing, and editing the article; YZ provided funding acquisition. All authors contributed to the article and approved the submitted version.
