# Supplementary figures and images for: Pan-cancer analysis of Krüppel-like factor 3 and its carcinogenesis in pancreatic cancer
Source: Front Immunol. 2023 Aug 3;14:1167018. doi: 10.3389/fimmu.2023.1167018 (PMC10435259; doi:10.3389/fimmu.2023.1167018)

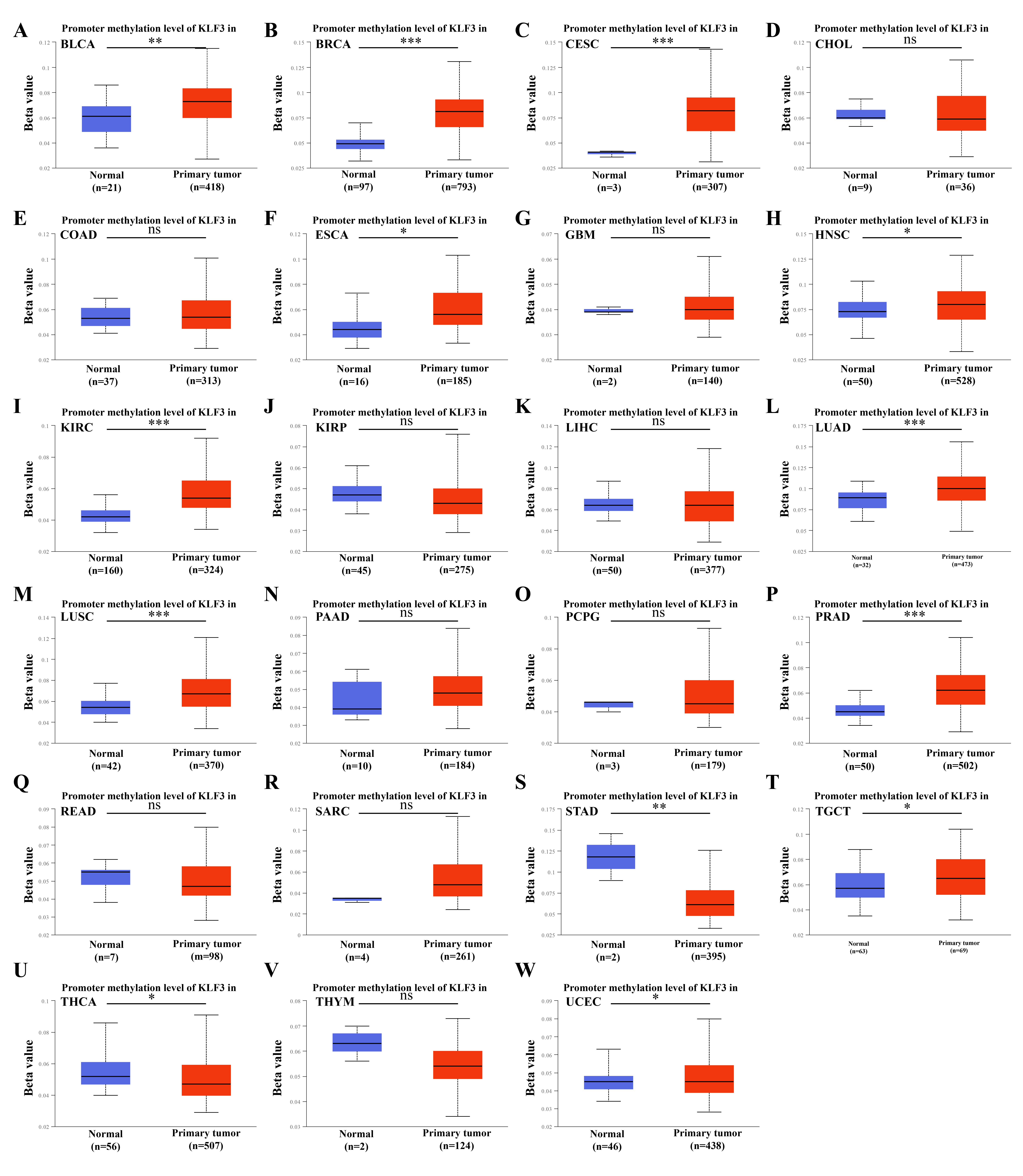

Supplement: Supplementary file 1 [file Image_1.tif]

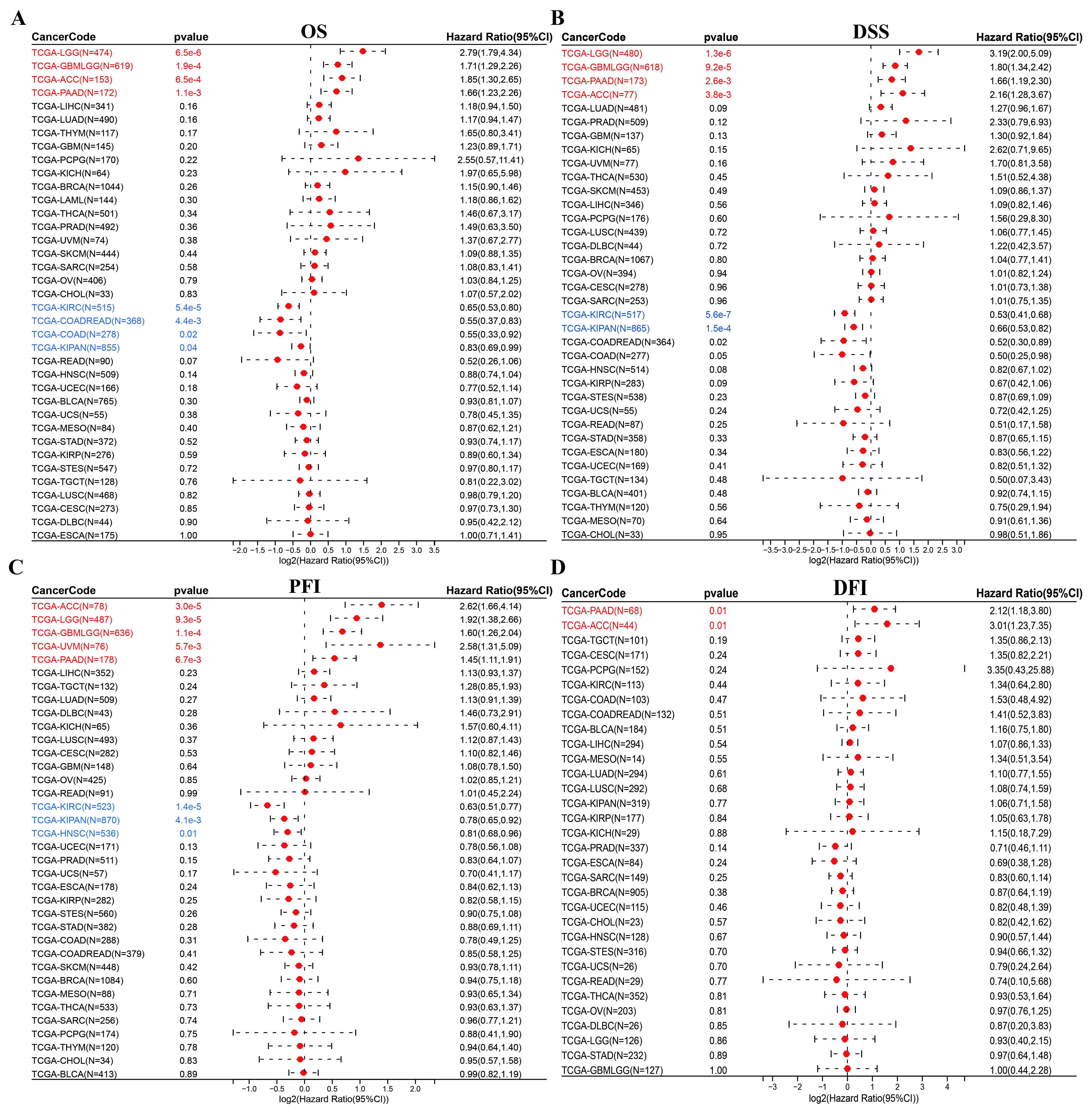

Supplement: Supplementary file 2 [file Image_2.tif]

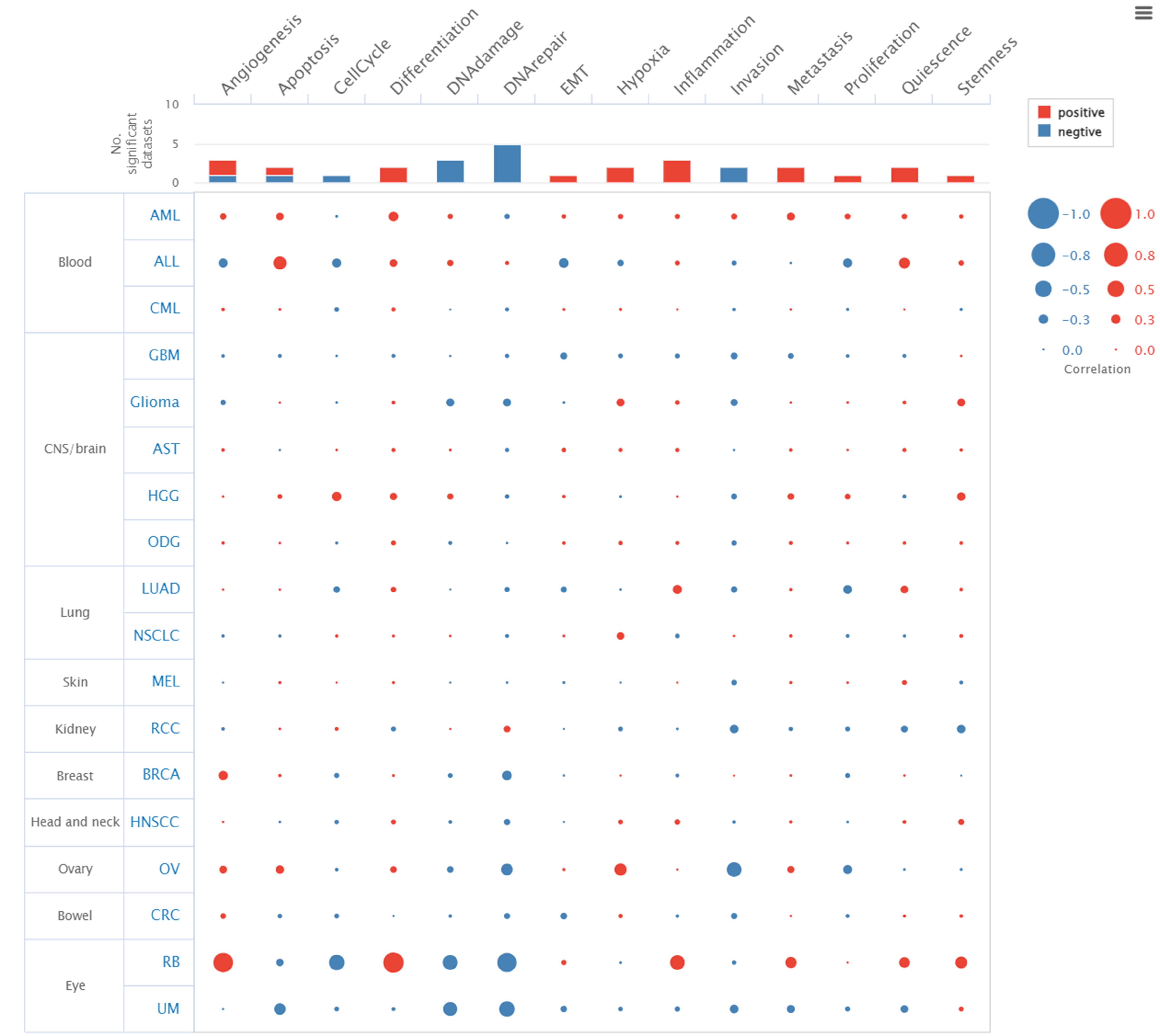

Supplement: Supplementary file 3 [file Image_3.tif]

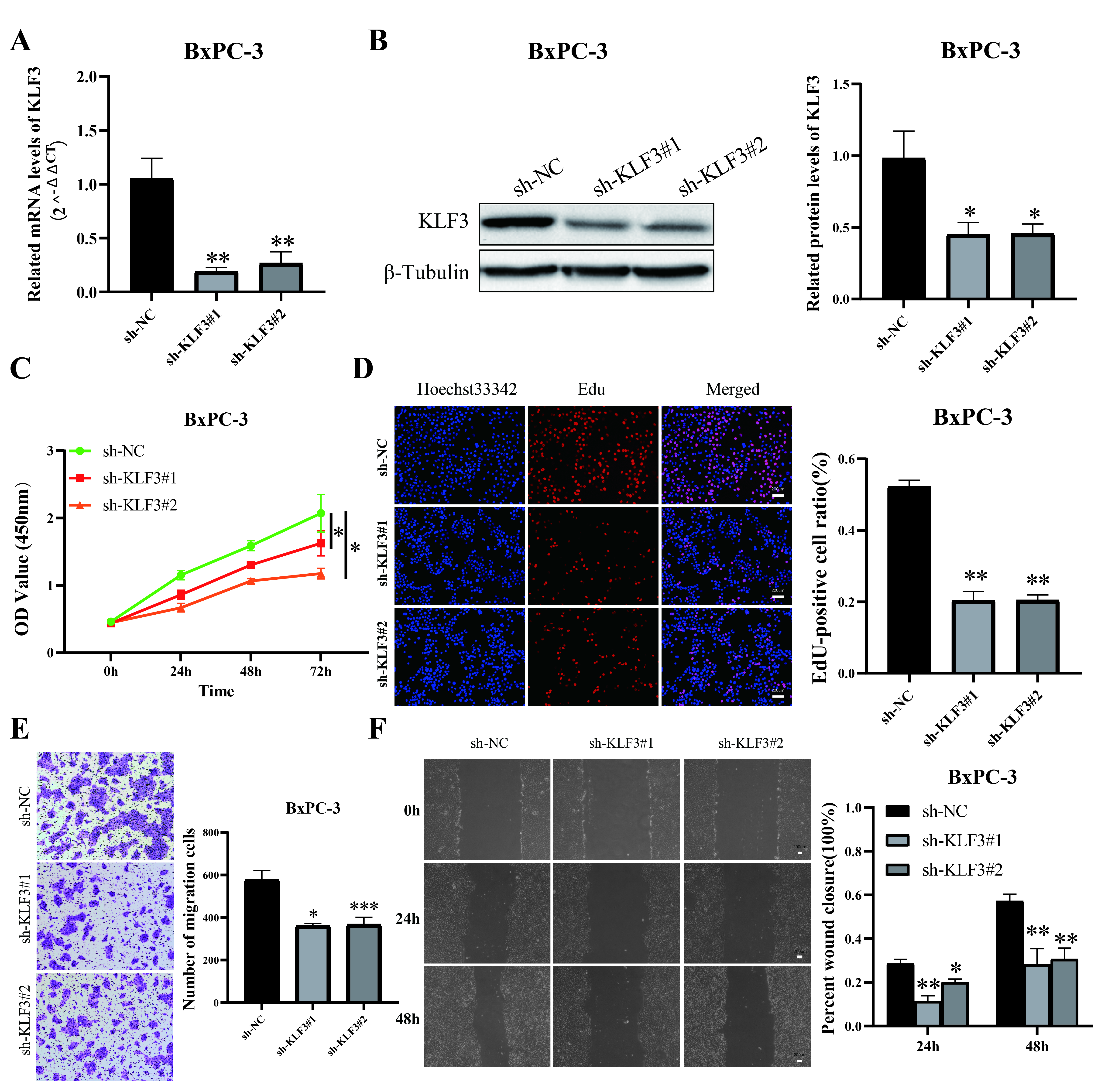

Supplement: Supplementary file 7 [file Image_7.tif]
