# Supplementary material for: Pan-cancer analysis of Krüppel-like factor 3 and its carcinogenesis in pancreatic cancer
Source: Front Immunol. 2023 Aug 3;14:1167018. doi: 10.3389/fimmu.2023.1167018 (PMC10435259; doi:10.3389/fimmu.2023.1167018)
Supplement: Supplementary file 8 [file DataSheet_1.docx]

*Supplementary Figure Legends*

Pan-cancer Analysis of Krüppel-Like Factor 3 and Its Carcinogenesis in Pancreatic Cancer

Jinfeng Zhu^1, †^, Hong Teng^1, 2, 3, †^, Xiaojian Zhu^4^, Jingxuan Yuan^1, 2, 3^, Qiong Zhang^1, 2, 3^ and Yeqing Zou^1, 2, 3*^

^1^Jiangxi Province Key Laboratory of Molecular Medicine, The Second Affiliated Hospital of Nanchang University, Nanchang, Jiangxi, China

^2^Department of Medical Genetics, the Second Affiliated Hospital of Nanchang University, Nanchang, China

^3^School of Public Health, Nanchang University, Nanchang, Jiangxi, China

^4^Tomas Lindahl Nobel Laureate Laboratory, The Seventh Affiliated Hospital of Sun Yat-sen University, Shenzhen, China

**^†^**Jinfeng Zhu and Hong Teng contributed equally to this work.

*** Correspondence:**

Yeqing Zou, Email：[zouyeqing3366@163.com](mailto:zouyeqing3366@163.com)

**Supplementary Figure Legends**

**SUPPLEMENTARY FIGURE 1.** DNA methylation level of *KLF3* gene promoter in pan-cancer.

(A-W) *KLF3* gene promoter DNA methylation level changes in corresponding normal tissues and BLCA, BRCA, CESC, CHOL, COAD, ESCA, GBM, HNSC, KIRC, KIRP, LIHC, LUAD, LUSC, PAAD, PCPG, PRAD, READ, SARC, STAD, TGCT, THCA, THYM and UCEC through the UALCAN database. *p < 0.05, **p < 0.01, ***p < 0.001, ns represents no significance.

**SUPPLEMENTARY FIGURE 2.** Effect of *KLF3* expression on prognosis of pan-cancer.

(A-D) Cox proportional hazards regression models were used to evaluate the association of *KLF3* expression with overall survival (OS) (A), disease-specific survival (DSS) (B), progression-free interval (PFI) (C), and disease-free interval (DFI) (D). Red label: prognostic risk factors, blue label: prognostic protection factors.

**SUPPLEMENTARY FIGURE 3.** Correlation between *KLF3* expression and functional status of cancer

Based on the single cell sequence data in the CancerSEA database, the correlation between *KLF3* expression and the functional status of 14 cases of cancer was analyzed.

**SUPPLEMENTARY FIGURE 4.** Relationship between *KLF3* expression and T-cell exhaustion, M2 macrophage and CAFs-related marker genes in pan-cancer.

**SUPPLEMENTARY FIGURE 5.** Immunotherapy response of *KLF3* in the immunotherapy group.

**SUPPLEMENTARY FIGURE 6.** Biomarker comparison of *KLF3* in the immunotherapy group.

**SUPPLEMENTARY FIGURE 7.** Silencing *KLF3* expression inhibits the progression of PAAD *in vitro*.

(A-B) qPCR and Western blot were used to detect the changes of *KLF3* mRNA and protein expression in BxPC-3 cells transfected with sh-*KLF3* plasmid. (C) CCK-8 method was used to detect the changes in the viability of BxPC-3 cells after transfection of sh-NC, sh-*KLF3*#1, and sh-*KLF3*#2 plasmids. (D) EdU staining was used to evaluate the changes in the proliferation ability of BxPC-3 cells in sh-NC, sh-*KLF3*#1, and sh-*KLF3*#2 groups. Transwell assay (E) and wound healing assay (F) were used to evaluate the changes in the cell migration ability of BxPC-3 cells in sh-NC, sh-*KLF3*#1, and sh-*KLF3*#2 groups. *p < 0.05, **p < 0.01, ***p < 0.001.
